# The role of social and ecological processes in structuring animal populations: a case study from automated tracking of wild birds

**DOI:** 10.1098/rsos.150057

**Published:** 2015-04-22

**Authors:** Damien R. Farine, Josh A. Firth, Lucy M. Aplin, Ross A. Crates, Antica Culina, Colin J. Garroway, Camilla A. Hinde, Lindall R. Kidd, Nicole D. Milligan, Ioannis Psorakis, Reinder Radersma, Brecht Verhelst, Bernhard Voelkl, Ben C. Sheldon

**Affiliations:** 1 Edward Grey Institute of Field Ornithology, Department of Zoology, University of Oxford, South Parks Road, Oxford OX1 3PS, UK; 2 Department of Anthropology (Evolution Wing), University of California, Davis, CA 95616, USA; 3 Smithsonian Tropical Research Institute, Ancon, Panama; 4 Research School of Biology, Australian National University, Acton 0200, Australia; 5 Behavioural Ecology Group, Department of Animal Sciences, Wageningen University, Wageningen, The Netherlands; 6 Pattern Analysis and Machine Learning Research Group, University of Oxford, Oxford, UK

**Keywords:** dispersal, great tit, group living, immigration, paridae, social organization

## Abstract

Both social and ecological factors influence population process and structure, with resultant consequences for phenotypic selection on individuals. Understanding the scale and relative contribution of these two factors is thus a central aim in evolutionary ecology. In this study, we develop a framework using null models to identify the social and spatial patterns that contribute to phenotypic structure in a wild population of songbirds. We used automated technologies to track 1053 individuals that formed 73 737 groups from which we inferred a social network. Our framework identified that both social and spatial drivers contributed to assortment in the network. In particular, groups had a more even sex ratio than expected and exhibited a consistent age structure that suggested local association preferences, such as preferential attachment or avoidance. By contrast, recent immigrants were spatially partitioned from locally born individuals, suggesting differential dispersal strategies by phenotype. Our results highlight how different scales of social decision-making, ranging from post-natal dispersal settlement to fission–fusion dynamics, can interact to drive phenotypic structure in animal populations.

## 2. Introduction

Group dynamics are an important part of an individual's social landscape. Group size can impact predation risk through dilution [1], selfish herd dynamics [2] or predator confusion effects [3]. The social links between individuals can also be important for gathering information about the environment [4–7], such as finding food [8–10]. Group-living often is typified by a trade-off between these benefits and costs incurred through competition [1]. However, costs and benefits of joining groups may vary according to the environment [11], individual phenotypic characters (such as those influenced by dominance or sex [12]) or with the existing composition of the group (similarity or difference in phenotype, for example the oddity effect [3]). If the benefits of being in a group vary with the phenotypes of its members, we should then expect regular patterns of associations to emerge between phenotypes in a way that maximizes the individual fitness of participants (such as kin structure in cooperative breeders [13]). How these patterns emerge in populations, whether from social (attraction or avoidance) or spatial (acceptance or exclusion) effects remains largely unexplored [14].

Fission–fusion societies are common across taxonomic groups. These are typified by groups that have short-term structural stability and high turnover in membership. Classic examples of animals that exhibit this social dynamic include primates [15–17], bats [18,19], elephants [20,21], red deer [22], as well as birds [23–26]. This dynamic structure is thought to result in linkages across all levels of these populations, from pairwise to landscape community interactions [27]. Such behaviour could be adaptive if simple pairwise interactions facilitate the emergence of complex patterns at the population scale [28,29]. For example, larger groups may be better at tracking environmental gradients [30]; hus, as the environmental signal deteriorates, shifting individual preferences towards a more gregarious joining policy can lead to group sizes best suited for current conditions [11]. In particular, fission–fusion dynamics are thought to facilitate behavioural plasticity, which could play a critical role in successful individual responses to environmental variability.

Individuals may also be able to influence particular selection pressures by choosing their social environment [31]. For example, males with poor sexual ornamentation could associate with other poor-quality males, enabling them to increase their relative quality, and subsequently benefitting their fitness [32]. Given that fission–fusion dynamics vary the membership of individuals across groups, the opportunities for these individuals to find an optimal social niche may be markedly higher in these systems than in species with more stable social systems. In this case, although the identities that individuals associate with in groups can change, the phenotypes of their associates may follow consistent non-random patterns [33]. For example, large fish may consistently interact with other large fish. Thus, unlike mechanisms relying on repeated interactions between individuals, such as cooperation [34], selection could influence individual fitness, regardless of the specific identity of participants in the groups [35].

In order to determine at what scale structured interactions between phenotypes (such as assortment or disassortment) could be operating, we recorded the composition of naturally occurring flocks of birds in a winter woodland population. Using a large dataset collected through extensive sampling of individuals fitted with passive integrated transponder (PIT) tags, we determine (i) how stable groups are over time, (ii) how groups varied in size according to population density and time of year, (iii) whether group composition reflected the local availability of individuals, and finally (iv) whether social or spatial variation in the distribution of phenotypes led to phenotypic assortment over the length of an entire winter. In doing so, we investigate the potential for social dynamics (i.e. group formation) to contribute to evolutionary processes, as non-random association of phenotypes can lead to variable selection [33].

## 3. Material and methods

The study took place at Wytham Woods, Oxford (51°46′ N, 1°20′ W). The breeding great tits (*Parus major*) in this 385 ha woodland are the focus of a long-term study, with over 1000 nest-boxes monitored annually. Since 2007, all breeding adults and chicks have been caught and fitted with PIT tags, in addition to the standard British Trust for Ornithology metal ring. Morphometric measurements, including age and sex were recorded for every bird caught as adults (93% of all birds in this study). This marking protocol has been supplemented by intensive autumn and regular winter catching, in order to ring and tag immigrant birds, as part of a study into their social ecology (see [8,36,37]). This approach enabled us to maintain in excess of 90% of the population fitted with PIT tags [23]. Recent studies suggest that social network analysis is robust against biases that may arise from small proportions of untagged individuals [38].

### 3.1 Field observations

While pairs of great tits maintain breeding territories during the spring, these dissolve post-breeding and the population structure turns into loose fission–fusion groups of predominantly unrelated individuals that roam in search of ephemeral and patchy food sources. In order to sample the social and phenotypic structure of these groups, we deployed 65 automated feeding stations in a stratified grid from 3 December 2011 to 26 February 2012. Each feeding station was fitted with two radio frequency identification antennae (one on each access hole) and filled with sunflower seeds. These were automatically opened for all daylight hours for 2 days per week, providing synchronous snapshots of the association patterns in the population. Feeders were filled with sunflower seed in order to minimize queuing (and therefore competition) as individuals picked up a seed and processed these in a nearby bush or tree. These feeders also maintained a constant reward, therefore removing any effects of patch depletion or developing differences between nearby feeding stations. Feeders scanned for PIT tags every one-third of a second from pre-dawn until after dusk and detected more than 99% of tagged individuals' visits to feeders. In all, we collected 26 days of data over 13 sampling periods.

### 3.2 Inferring group membership

Feeding stations provided a highly resolved spatio-temporal data stream of individual visits. As individuals fed in groups, or flocks, the pattern of visits typically contained bursts of high activity, separated by periods of low activity. Given the stochastic nature of this system (groups may feed for different lengths of time), we inferred group membership using a machine learning algorithm based on Gaussian mixture models [39]. This avoids the need to impose arbitrary temporal boundaries on groups. Instead, it infers the best-fitting window for each group based on the patterns observed over the entire dataset, resulting in a more accurate social network than other approaches [40]. By fitting a Gaussian distribution over closely spaced visits, visits can then be assigned to a burst, or group, to which they have the highest probability of belonging. This method returns a matrix of groups and the individual's membership of these groups.

### 3.3 Stability

We used a measure of temporal group stability that is similar to the lagged rate of association proposed by Farine [41]. This measure represents the proportion of individuals that are consistent across two groups containing a focal individual *X* and separated by a time period *τ*, given by

$$S(\tau ,X)=\frac{1}{G_{\tau }}\sum_{j,k|(t_{k}-t_{j})=\tau }\frac{G_{j,k}(X)}{G_{j,!k}(X)+G_{k,!j}(X)+G_{j,k}(X)},$$

where *G*_*j*_(*X*) is the number of occurrences of groups containing focal individual *X* and split by *τ*, *G*_*j*,*k*_(*X*) is the number of non-focal individuals occurring in two groups, both containing individual *X*, and separated by *τ*=*t*_*k*_−*t*_*j*_. *G*_*j*,!*k*_(*X*) is the number of individuals occurring only in group *j*, and *G*_*k*,!*j*_(*X*) is the number of individuals occurring only in group *k*. *S*(*τ*,*X*)=0.5 is equivalent to a group fusing with another group twice the size, or if two-thirds of a group remains the same in two evenly sized groups, over a time period *τ*. We limited this calculation to groups that contained at least one common member in order to ensure that there was a common link between groups. To estimate how the observed stability differed from random, we calculated *S*(*τ*,*X*) for groups in a spatio-temporally restricted null model (see below). This model constrains the distribution of group sizes and uses the ratio of the observed to permuted data to estimate the period *τ* in which non-random associations persist.

### 3.4 Group composition

When investigating how group composition changed over different group sizes, it was necessary to pool certain groups together when calculating test statistics (e.g. ‘mean sex assortativity’), due to low sample sizes for larger groups. Therefore, group sizes larger than 13 were binned into ‘group size classes’ (e.g. [14,15]) that contained at least 5% of the total group memberships (figures 3–5).

### 3.5 Within-group membership

We explored how the composition of the groups varied with regard to group size for three different state variables (sex, age and residency status), all of which are binary states (male or female; juvenile or adult; immigrant or locally born, respectively). Sex was determined when birds were recaptured (93% of individuals were sexed) using plumage characteristics (the width of the breast stripe is much larger in males). Juveniles are defined as birds that were born in the breeding season immediately prior to the winter (in this case in spring 2011). Immigrants are defined as birds that were born outside of the study area (dispersed into Wytham Woods), whereas locally born birds were ringed as nestlings within the study area (the proportion of nestlings born in natural cavities is estimated to be very low [42]). The demographic structures of groups were calculated as the proportion of individuals from each class occurring in each separate group, and pooling these data as the mean for each group size. We then compared these means to groups in permuted data (described below).

Further, we also examined whether group size was associated with the body size of individuals within them. As a multivariate measure of size for individuals, the first principal component (PC1) of a principal components analysis using wing and tarsus length (available for 84% of all individuals) was used. As males were significantly larger in both measures (*t*-test; wing: *t*=28.82, d.f.=931, *p*<0.001, tarsus: *t*=13.63, d.f.=885, *p*<0.001), this measure was generated separately within each sex and standardized with a mean of 0 with a variance of 1. PC1 correlated strongly with both size measures in both sexes (Pearson's correlation coefficient; male wing=0.998, male tarsus=0.323, female wing=0.998, female tarsus=0.288). We then calculated the mean size of individuals within each age and sex class for each individual group, and again compared the mean of these values of each different group size to the permuted data.

### 3.6 Within-group assortativity

After determining the content of groups in regard to the different demographic states (above), within-group assortativity for each demographic state was examined separately. We calculated the binomial probability that, given the group size and the total number of unique individuals in each class over all groups of that group size, the observed number of individuals in these classes would have occurred by chance. For example, if among all the individuals who participated in groups of size two we had an equal number of males and females, a group containing two males (or two females) would be assigned 0.25, while a group containing one male and one female would have a value of 0.5. It is always the case that higher values (i.e. closer to 0.5) represent more disassortativity. Following this, an overall mean was calculated for these values for each group size and again compared to the permuted data to test for significance.

We also considered whether groups show assortativity by size, both overall and within the different demographic classes. This was determined by calculating the mean size for each group (and the mean size of the different classes of individuals within the group to make ‘within class’ comparisons). Then, for each group size, we obtained a kurtosis score for the distribution of these mean sizes (see [23]), where high kurtosis scores indicate a peaked distribution (i.e. assortativity), while low scores indicate a flat distribution (i.e. disassortativity).

### 3.7 Null models

We used randomization techniques in order to create two contrasting null models. The first assumed random interactions between individuals but a fixed observation stream, which we call the phenotypic null model. In this method, we created 1000 random networks by shuffling the node labels associated with each individual's phenotype. The second maintained spatio-temporal distributions of phenotypes; we call this the spatio-temporally controlled null model. These two randomizations used two general methods of data randomizations. Phenotypic randomizations consisted of randomly re-allocating the phenotype of individuals, maintaining the same observation patterns and the same distribution of phenotypes. Spatio-temporally controlled randomizations used a restricted permutation test following the methods originally described by Bejder *et al*. [43] with subsequent improvements by numerous authors (see [44], pp. 125–127). This null model randomly swaps the observations of two individuals observed in different groups, with swaps being restricted to control for space and time. Each step in the permutation performs one of these swaps, creating an increasingly random dataset. In our case, we restricted swaps to occur only between groups that were observed at the same location (same feeder) and on the same sampling period (weekend). The resulting output is a data stream where the size (and time and location data) of each group remains constant along with the number of observations for each individual, but the detailed patterns of group membership are changed. This therefore tests whether structure exists within each location given the variation in the number of observations for each individual.

These two null models allowed us to partially differentiate between patterns arising from spatial and social effects [45]. If the patterns between the two randomizations differ, this suggests that phenotypes are not evenly distributed in space. This is because spatial variation in the number of individuals of each phenotype is maintained in the spatio-temporally controlled null model, but not in the phenotypic null model. If the observed data then match the spatio-temporal but not the phenotypic null model, then any assortment in this phenotype is attributable to spatial effects. If the data differ from both, then we attribute assortment to be due to social effects given that phenotypes are randomly distributed in space.

Comparing the observed patterns of group membership with null models derived from permutation allowed us to reduce the potential impact of pseudoreplication in our data. Both the null models maintained the same underlying structure of interactions, such as group sizes, number of observations for each individual and the temporal properties of group structure (group size increases as a proportion of time of day [36]). Although our data comprise many repeated observations of the same individuals and occasionally even the same groups, these repetitions are repeated in the permuted data. Taking the ratio of measures calculated on both observed and permuted data provides a measure of how non-randomly groups and occurrences are repeated.

We repeated the phenotypic randomization 1000 times. We ran 1 000 000 iterations of the spatio-temporal randomizations, as only a single observation is swapped on each iteration (whereas phenotypic randomizations shuffle all nodes; see [44], p. 130). Where possible, we compared our data to the distribution of the spatio-temporal randomizations (taken after every 1000th iteration), but where this was not possible (for example for the group stability measure that is highly computationally intensive) we used only the final randomized group membership matrix after the 1 000 000th iteration.

### 3.8 Social network analysis

We used the R [46] package *asnipe* [41] to calculate the simple-ratio association index between all interacting dyads. This index scales edge weights between 0 (never observed together) and 1 (always observed together). We then calculated the assortativity index [47], which is derived from the Pearson correlation coefficient for weighted-edge networks in the R package *assortnet* [14].

## 4. Results

We detected a total of 1053 individual great tits, consisting of 274 adult females, 252 adult males, 234 juvenile females, 229 juvenile males and 64 individuals of unknown sex (that were never caught post-fledging and not included in subsequent analyses). Across the 13 sampling periods, most individuals were detected on a large majority of the sampling periods (mean=9.4, median=11). Overall, we logged 3 347 038 unique detections of individuals over the 13 sampling periods, forming 73 737 unique groups with a mean size (±s.e.) of 4.7±0.01 individuals and a typical group size (i.e. group size experienced by the average individual, ±s.e.) of 7.5±0.01 individuals per group.

### 4.1 Stability of group membership

**Figure 1.**
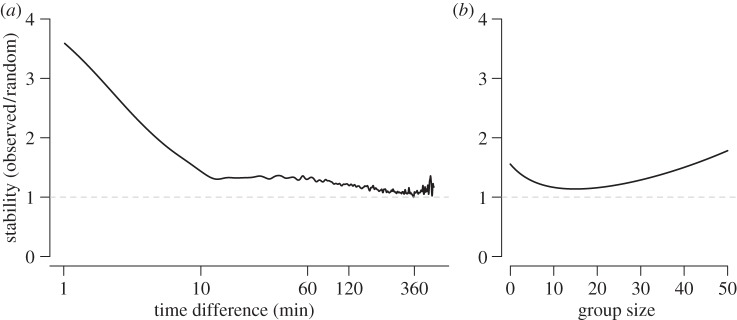
Groups show higher stability than expected from random, both in terms of (*a*) the (log) time-gap between groups and (*b*) the size of the initial group. Groups were most stable in the period of 1–10 min, after which stability was non-random, but much lower, up to approximately 2 h (see electronic supplementary material, figure S1, for independent curves). Small and large groups were the most stable relative to chance. Solid lines show the ratio of the observed stability to the stability calculated from randomized data.

We found that groups had significantly higher stability in the first 10 min after being observed when compared with the same data after 1 million randomizations of the spatially and temporally restricted null model (figure 1*a*). As the inter-group time interval increases, the difference between the observed stability and the stability of random groups approached zero. Group composition was therefore stable over short periods of time (less than 10 min), with only a few stable associations being maintained over longer periods (see also [23]). Stability also varied according to group size, with small and large groups being more stable than expected by chance (figure 1*b*).

### 4.2 Group size distributions

**Figure 2.**
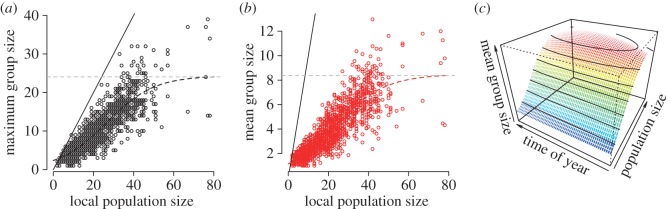
Group size was proportionately smaller as local population size increased. For each location, we calculated the number of individuals recorded at the site in each sampling period. We found that both (*a*) maximum group size and (*b*) average group size saturated with increasing population size. (*c*) We found no effect of season on this relationship, where time of year represents the period ranging from 3 December 2011 to 26 February 2012.

We found a strong divergence from a 1 : 1 relationship between the number of individuals present on a given day and the group sizes observed (figure 2*a*). Logistic models of the mean and maximum observed groups sizes had significantly greater support than linear models applied to the same data (electronic supplementary material, table S1). This suggests that the relationship between population size and mean or maximum group size saturated, in this case at a maximum of eight and 24 individuals per group, respectively (figure 2*a*,*b*, horizontal dashed lines). Further, the relationship between mean group size and population size appeared to remain rather stable between weeks over the winter (generalized linear mixed model controlling for population size with location as a random effect suggests a very weak decrease in group size each week: *t*=−1.85, *B*±s.e.=−0.012±0.006, *p*=0.06; figure 2*c*).

### 4.3 Group composition

Non-random distribution of individuals according to their phenotypes was found among groups, and this differed with group size. Non-random group composition can be driven by spatial distribution of phenotypes (inferred here from the observed data differing significantly from the ‘phenotypic randomization’ null model) or socially driven, i.e. active decisions by individuals regarding group membership (inferred here from the observed data differing significantly from the ‘spatio-temporal controlled’ null model, or both). Indeed, we found evidence for all three situations in regards to the demographic states considered here.

**Figure 3.**
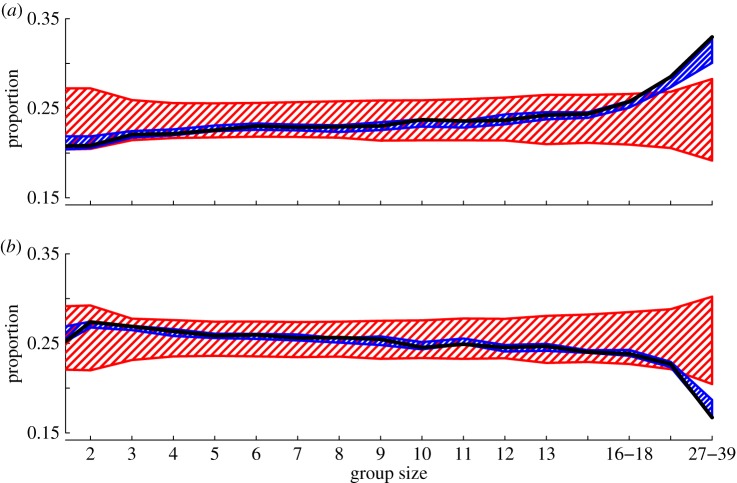
There was a significantly greater proportion of females in large groups that were juvenile (*a*) and lower proportion of males in large groups that were adults (*b*). The black lines show the observed data, the blue polygons show the 95% range of permutation data from the ‘spatio-temporally controlled null model’, and the red polygons show the 95% range of permutation data generated from the ‘phenotypic randomization null model’. In groups of over 18 individuals, the observed proportions of the age classes of males and females in each differ from the phenotypic randomization null model only. This suggests that juvenile females were disproportionately found in areas with large flocks, whereas adult males were absent from areas containing large flocks.

Although groups of all sizes contained the expected proportions of adult females and juvenile males (electronic supplementary material, figure S2), we found that large groups contained more juvenile females but fewer adult males than expected by the phenotypic randomization model (figure 3). These comparisons were carried out simultaneously over multiple bins, and although standard Bonferroni corrections are not appropriate here due to the non-independence of the data, we found that, for the largest group sizes, the observed proportion of juvenile females and adult males fell outside of the entire range of the data generated from phenotypic randomization. As the spatio-temporal null model matched the observed data (figure 3), this suggests a spatially driven pattern, such that areas containing large groups also contain higher proportions of juvenile females in comparison to adult males.

**Figure 4.**
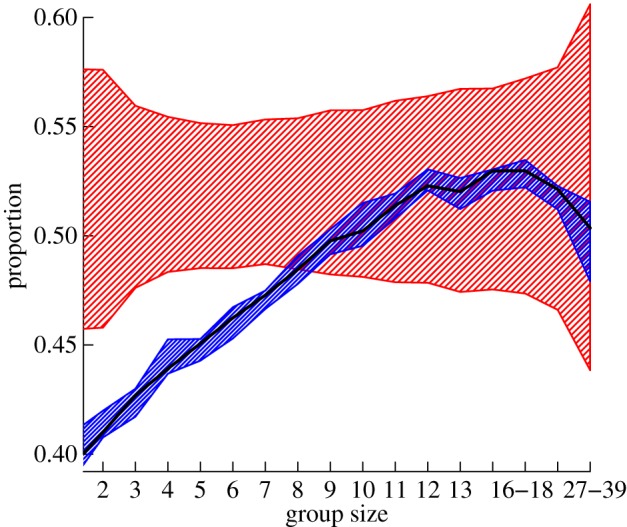
There was a significantly smaller proportion of immigrant juveniles in groups of less than 8. The observed data (black line) differ significantly from the phenotypic randomization null model (red polygon), but not the spatio-temporally controlled model (blue polygon), suggesting that immigrant juveniles are found in areas of Wytham Woods where large flocks occur.

A similar pattern was also found for the proportion of juveniles that were immigrants in small groups (less than 8), which was significantly lower than the phenotypic randomization null model but predicted very well by spatio-temporally controlled permutations (figure 4), suggesting a non-uniform distribution of juvenile immigrants across the study area. However, no such pattern of non-random group content based on residency status was observed among adult birds (electronic supplementary material, figure S3).

**Figure 5.**
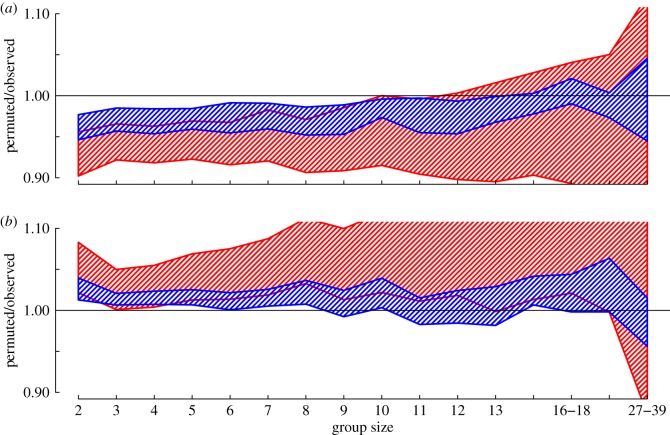
Groups were significantly (*a*) more evenly distributed by sex but (*b*) more assorted by age class than expected from the randomized datasets. In these plots, the *y*-axis shows the ratio of the null models to the observed data (observed data are *y*=1). The phenotypic null model is shown by the red polygon and the spatio-temporally controlled null model is shown by the blue polygon. The sex ratio of groups up to a size of 10 is significantly closer to even (observed is more than random) than both the spatio-temporally restricted and the phenotypic randomizations (see electronic supplementary material, figure S6, for these shown as binomial probabilities). These groups also tend to contain more individuals that are either adults or juveniles than expected (groups are significantly less mixed than expected, observed is less than random). Plots show the ratio of the 95% confidence intervals of the randomizations to the observed data. Areas where the polygon overlap 1 are non-significant (the permutation is equal to the observed value). Plots are presented this way due to the difficultly of directly interpreting binomial probabilities, and the biological insignificance of the exponential decay as group size increases (see electronic supplementary material, figure S6).

As well as the proportion of individuals of different phenotypes included in groups, we also found non-random assortment in regards to different demographic characteristics. Groups were found to be more evenly distributed with regard to sex (i.e. sex ratio closer to equal) than expected by both null models, which also fall within each other's range, thus suggesting a primarily socially driven pattern (figure 5*a*). By contrast, groups tended to show assortativity by age, as the proportion of either adults or juveniles in each group was higher than expected by chance (figure 5*b*), and again appeared largely driven by local social group structure rather than variation due to spatial disaggregation.

The mean size of individuals within groups did not differ from either the spatial or population-wide distributions of phenotypes (electronic supplementary material, figure S4). This was true within all demographic classes of individuals, apart from a slight trend for larger juveniles than expected by the node permutation model in mid-size groups (electronic supplementary material, figure S4). We also found no major patterns of phenotypic assortment in groups according to individual size among any of the different demographic classes (electronic supplementary material, figure S5), suggesting that these did not differ dramatically from either the spatial or population-wide distributions of phenotypes.

### 4.4 Assortment in the social network

The social network contained a single fully connected component, with a link density of 0.08 (8% of possible dyadic edges are present). These edges were significantly disassorted by sex (assortativity coefficient *r*=−0.034±0.0009), which indicates that mixed-sex dyads had a higher probability of both occurring and reoccurring. Age (in years) was significant assorted (*r*=0.047±0.0014), suggesting that individuals of the same type, particularly juveniles, had a stronger tendency of repeatedly associating during the winter. Finally, we found significant overall assortment by immigration status (*r*=0.037±0.0019), which was mostly driven by assortment in adults (*r*=0.11±0.0034).

## 5. Discussion

We used an automated detection system to collect data on the patterns of association between individuals in a large free-ranging bird population to understand how individuals of different types associate and what mechanisms produce non-randomness in association. We showed that although groups were highly unstable in membership over short periods of time, interactions between different phenotypes in the population were not themselves random. In some cases, our results suggest that the patterns of group membership arose through association preferences by individuals, potentially based on both their own phenotypes but also the existing composition of the group. In others, group structure appears to be shaped by the variation in the distribution of phenotypes across space. For example, adult males (which are often more dominant [48]) tended to avoid large groups, whereas juvenile females (generally subdominant phenotypes [49]) were found in larger groups more often than expected.

These findings have important implications: (i) individuals may have different social strategies as a function of their phenotypes and (ii) differences in short-term strategies can maintain long-term variation in interaction rates between phenotypes as measured in our winter-long social network. As a consequence, fission–fusion dynamics may influence the pressures of selection operating upon different phenotypes [35]. For example, in species such as great tits, where residency is an important predictor of dominance [48], associating with other immigrants may help reduce the overall competition a newly arrived individual experiences. This effect of mediating selection pressure by choosing with whom to associate (beyond the simple changes in group size [28]) may play an important role in shaping the evolution of this social system.

Although phenotypic assortment may be relatively common in nature, no study has, to our knowledge, linked short-term group membership dynamics to long-term association patterns in a social network. In fish, several studies have found that groups will assort by size (reviewed in [50]), often in preference to species identity [51,52]. Also, assorted group composition appears to play a particularly important role in avoiding predation, either by minimizing an individual's risk relative to the group [53] or by maintaining movement synchrony [54]. Body size assortment can also emerge simply through spatial variation in the distribution of phenotypes, for example when fish of different age classes (and therefore different sizes) inhabit different niches [55].

We found that groups were often more mixed by sex than expected from chance across a wide range of group sizes. This was almost certainly a result of social decisions, leading to significant disassortment over the entire study period. In socially monogamous birds, this is perhaps not unexpected. For example, Wilkinson [56] found that groups of bullfinches (*Pyrrhula pyrrhula*) were more mixed by sex than expected by chance. By contrast, brown-headed cowbirds (*Molothrus ater*) showed the strongest links between females within the fission–fusion social dynamics of that species, despite being brood parasites and having been raised in the nests of other species [57]. The authors concluded that assortment may play an important role in developing skills needed for breeding in that species [57].

Individuals in our study were also assorted with regard to their status as immigrant or locally born birds. This appeared to be driven by spatial sorting rather than social decisions in the flocks. Recent work in this population suggests that by associating with other recently arrived individuals, late arrivals could reduce the selection operating on their dispersal phenotype [35]. In parids, there is a strong interaction between residence and dominance, with birds that hold or have previously held a territory having relatively higher dominance than non-residents [48,58]. This may lead to spatial partitioning if resident birds exclude immigrants from core or high-quality zones, and these immigrants associate in marginal or bordering areas. This effect may even include resident juveniles being dominant over recently arrived adults [48]. Alternatively, immigrant birds may have greater similarities in their activity levels; for example, immigrants are typically more ‘bold’ along a spectrum of slow explorer to fast explorer [59,60], and consequently have been found to be more closely associated in this population [23].

Although these groups were very unstable in time, they maintained consistent relationships between and within particular phenotypes and maintained strikingly consistent group sizes regardless of season. Importantly, patterns of structure in group membership led to long-term assortment in the social network that were driven by both social and spatial mechanisms. Our findings suggest that the social decisions made by individuals on a day-to-day basis may have an important role in shaping the strength or direction of selection operating on different phenotypes, even in fission–fusion societies.

## Supplementary Material

Supplementary Table and Figures

## Data accessibility

Data are available to download from Dryad (http://dx.doi.org/10.5061/dryad.885c0).
