# Supplementary material for: The role of social and ecological processes in structuring animal populations: a case study from automated tracking of wild birds
Source: R Soc Open Sci. 2015 Apr 22;2(4):150057. doi: 10.1098/rsos.150057 (PMC4448873; doi:10.1098/rsos.150057)
Supplement: Supplementary Table and Figures [file rsos150057supp1.pdf]

***Farine et al*. The role of social and ecological processes in structuring animal populations: a case study from automated tracking of wild birds**

SUPPLEMENTARY TABLE

Table S1. Summary of AIC support for linear and logistic regressions of the relationship between group size and local population size.

Model	*AIC*
Max group size ~ Population	7052
Max group size ~ logis(Population)	7014
Mean group size ~ Population	4196
Mean group size ~ logis(Population)	4091

SUPPLEMENTARY FIGURES

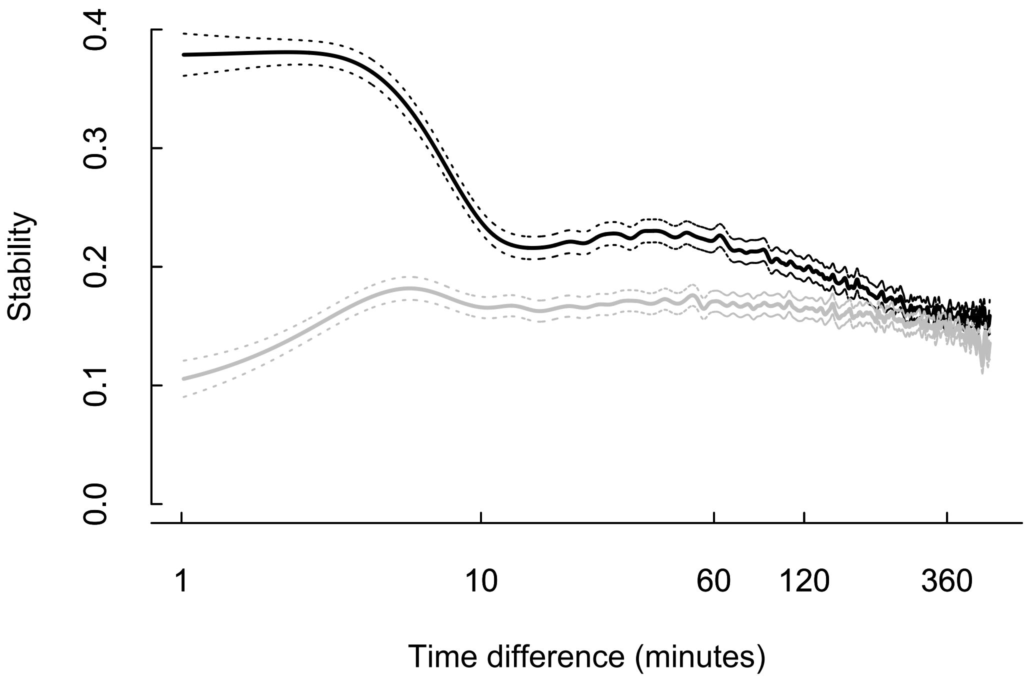

Figure S1: Group stability shown for the observed data (top black line) and randomised (bottom grey line) data after 1 000 000 iterations, shown with 95% confidence intervals calculated using jackknife.

(a)

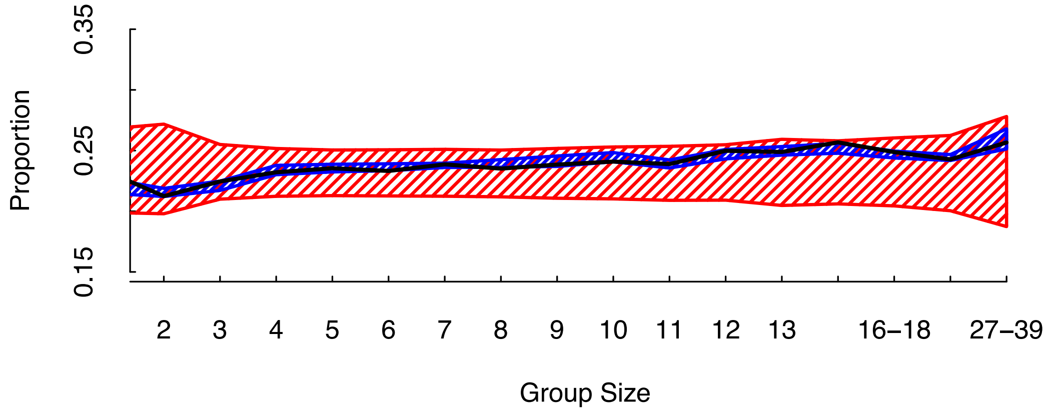

(b)

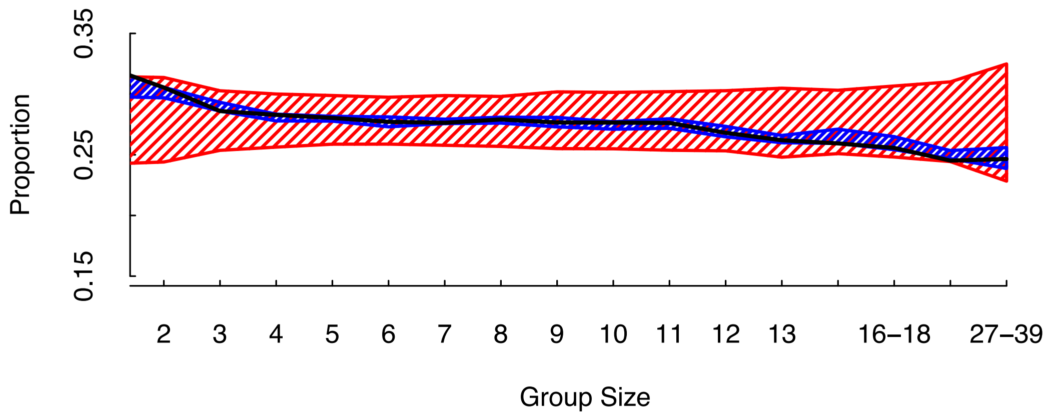

Figure S2: The proportion of a) Juvenile males & b) Adult females in the observed data match that expected from both the phenotypic randomisation null models and the spatiotemporal model.

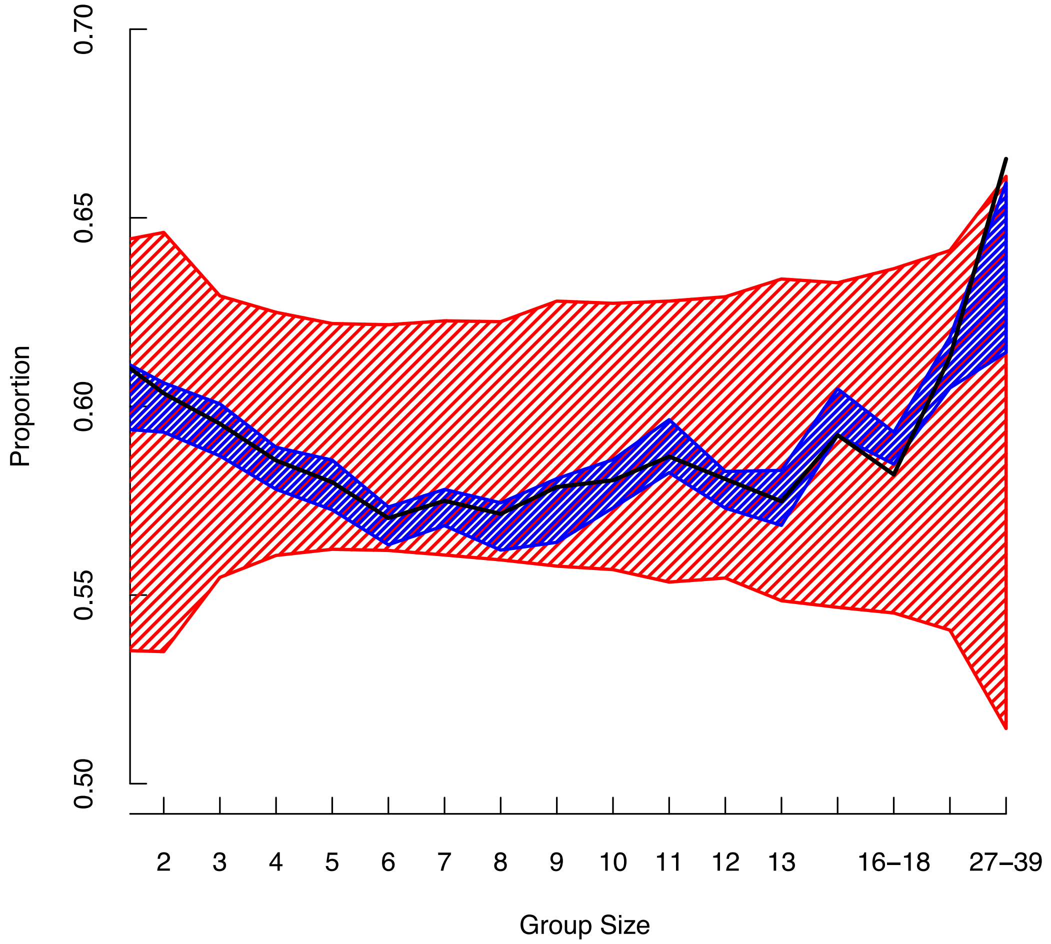

Figure S3: The proportion of adults that are immigrants in all groups matches that expected by both null models.

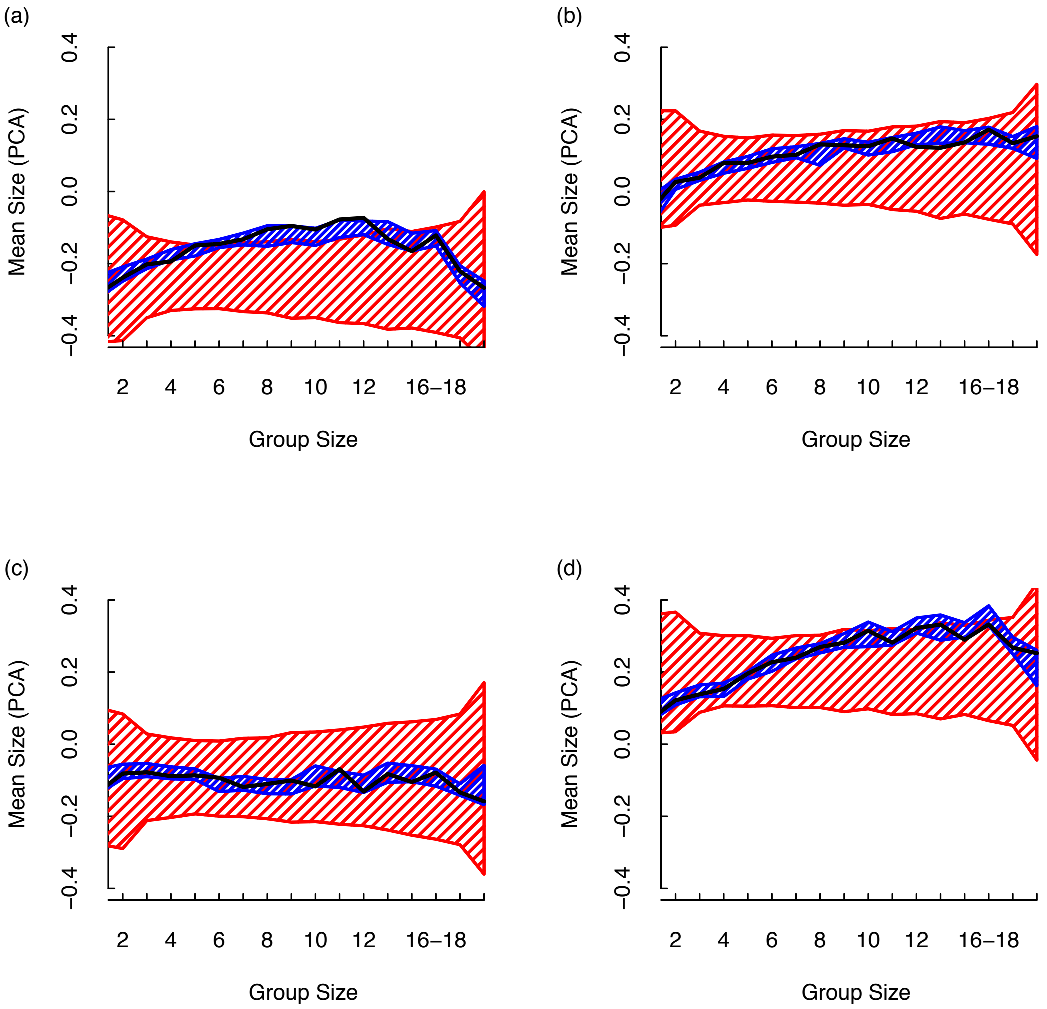

Figure S4: The mean size (PCA1 – see Methods) of individuals within a group for the four classes; (a) Juvenile Male, (b) Adult Female, (c) Juvenile Female, (d) Adult Male. In all cases, the observed data matches the null models, apart from JM, where mid-sized groups (6-12 individuals) contains individual's larger than that expected by the phenotypic randomisation model.

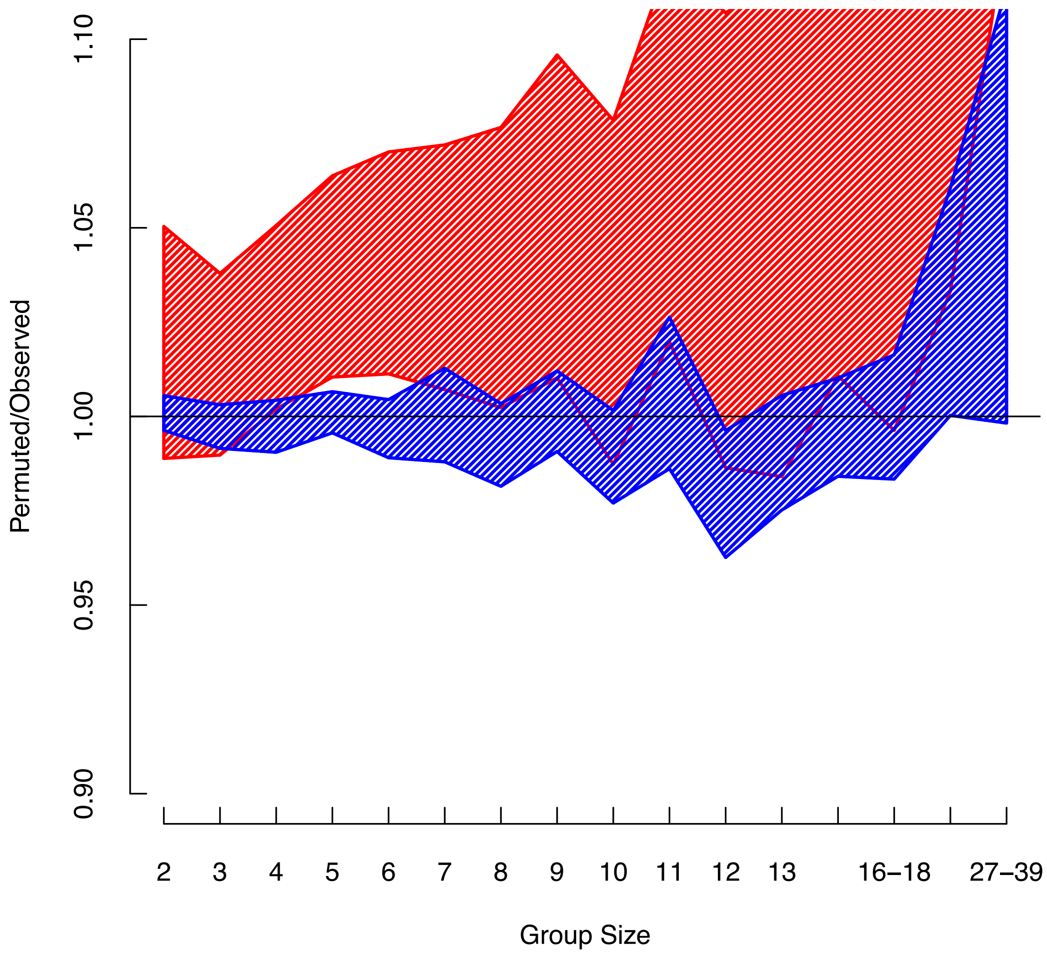

Figure S5: Assortativity for residency status. Over all group sizes, the observed data is very similar to the spatiotemporally controlled null model. However, the observed residency assortativity for groups between 4-9 individuals appeared higher than that expected by the phenotypic randomisation model.

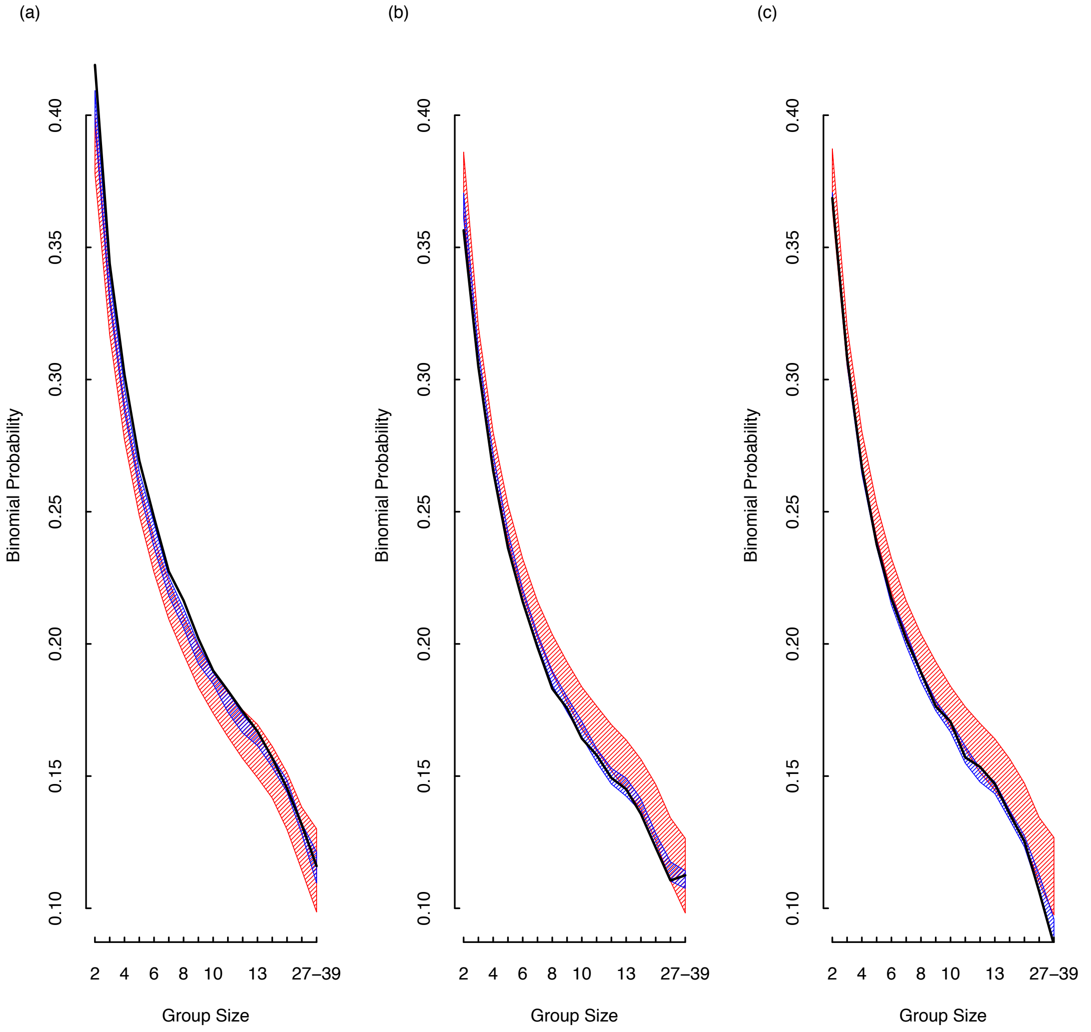

Figure S6: Observed, spatiotemporal controlled null model, and phenotypically random null model binomial probabilities (i.e. Assortativity index) for a) Sex, b) Age and c) Residency Status, over the different group sizes. This data is presented in the manuscript as Permuted/Observed values, due to the biological insignificance of the exponential decay of the binomial probability with group size, and the difficultly of easily interpreting such data.
